# The *TREM2* H157Y Variant Influences Microglial Phagocytosis, Polarization, and Inflammatory Cytokine Release

**DOI:** 10.3390/brainsci13040642

**Published:** 2023-04-09

**Authors:** Xin-Xin Fu, Shuai-Yu Chen, Hui-Wen Lian, Yang Deng, Rui Duan, Ying-Dong Zhang, Teng Jiang

**Affiliations:** 1 Department of Neurology, Nanjing First Hospital, China Pharmaceutical University, No.639 Longmian Road, Nanjing 211100, China; 2 Department of Neurology, Nanjing First Hospital, Nanjing Medical University, No.68 Changle Road, Nanjing 210006, China

**Keywords:** Alzheimer’s disease, *TREM2*, H157Y variant, amyloid-β, CRISPR-Cas9, microglia

## Abstract

Previously, we reported that H157Y, a rare coding variant on exon 3 of the triggering receptor expressed on myeloid cells 2 gene (*TREM2*), was associated with Alzheimer’s disease (AD) risk in a Han Chinese population. To date, how this variant increases AD risk has remained unclear. In this study, using CRISPR-Cas9-engineered BV2 microglia, we tried to investigate the influence of the *Trem2* H157Y variant on AD-related microglial functions. For the first time, we revealed that the *Trem2* H157Y variant inhibits microglial phagocytosis of amyloid-β, promotes M1-type polarization of microglia, and facilitates microglial release of inflammatory cytokines, including interleukin (IL)-1β, IL-6, and tumor necrosis factor-α. These findings provide new insights into the cellular mechanisms by which the *TREM2* H157Y variant elevates the risk of AD.

## 1. Introduction

Currently, Alzheimer’s disease (AD) is the most common type of neurodegenerative disorder in the elderly population [1]. It is characterized by progressive decline in cognition, including memory, decision making, and linguistic functions [2]. The pathological features of AD include amyloid-β (Aβ) deposition, tau hyperphosphorylation, loss of neurons and synapses, and neuroinflammation [3]. However, the etiology and pathogenesis of AD remain elusive.

Accumulating evidence suggests that activation of microglia contributes to the pathogenesis of AD [4]. On the one hand, activated microglia participate in the phagocytosis of Aβ, thus preventing amyloid plaque formation [5]. On the other hand, long-term activation of microglia leads to release of inflammatory cytokines, which subsequently causes bystander neuronal and synaptic injuries [6]. Several lines of evidence have indicated that multiple important functions of microglia, including phagocytosis, polarization, and release of cytokines, are tightly regulated by several immune receptors, such as triggering receptor expressed on myeloid cells 2 (TREM2) [7,8,9,10,11]. *TREM2* is located on human chromosome 6p21, a hot zone linked with AD susceptibility [12,13]. We previously revealed that H157Y, a coding variant on exon 3 of *TREM2*, was associated with AD risk in a Han Chinese population [14,15]. To date, how this variant increases AD risk has not been fully understood.

In this study, using CRISPR-Cas9-engineered BV2 microglia, we tried to investigate the influence of the *Trem2* H157Y variant on AD-related microglial functions. For the first time, we revealed that the *Trem2* H157Y variant inhibits microglial phagocytosis of Aβ, promotes M1-type polarization of microglia, and facilitates microglial release of inflammatory cytokines. These findings provide insights into the cellular mechanism by which the *Trem2* H157Y variant elevates the risk of AD.

## 2. Methods

### 2.1. CRISPR-Cas9-Mediated Trem2 H157Y Variant Knock-In

BV2 mouse microglia with the *Trem2* H157Y variant were generated using CRISPR-Cas9 technology. Based on the genomic sequence of mouse *Trem2*, gRNA was designed to target the regions near the variant site [16]. Cleavage efficiency was estimated by sequencing trace analysis with online tools [17,18]. A donor template containing the wild type (WT) C or variant T allele on gRNA recognition sites was designed. *Trem2* was mutated by co-transfection of plasmids containing the gRNA and Cas9. The transfected BV2 microglia were plated in 96-well plates by limit dilution to generate isogenic single clones. The clones were selected from wells and screened by restriction endonuclease digestion and DNA sequencing.

### 2.2. BV2 Microglia Culture and LPS Stimulation

BV2 microglia were cultured in DMEM medium (Thermo Fisher Scientific, Inc., Waltham, MA, USA) containing 10% fetal bovine serum (Thermo Fisher Scientific, Inc., Waltham, MA, USA) and 1% penicillin-streptomycin solution (Thermo Fisher Scientific, Inc., Waltham, MA, USA). To induce microglial inflammatory response, BV2 microglia were stimulated with 100 ng/mL lipopolysaccharide (LPS) diluted in DMEM medium at 37 °C for 24 h, as described previously [19,20].

### 2.3. Aβ Phagocytosis and Degradation Assays

An in vitro assay was employed to evaluate microglial Aβ_1–42_ phagocytosis and degradation, as described previously [21,22]. Aβ_1–42_ (AnaSpec, Inc., Liege, Belgium) was dissolved in phosphate-buffered saline (PBS) at a concentration of 1 mM. To measure the ability for Aβ_1–42_ phagocytosis, BV2 microglia were incubated with 5 μM Aβ_1–42_ for 6 h. Cells were then washed using PBS and trypsinized to remove surface-bound Aβ_1–42_, followed by lysis. The amount of internalized Aβ_1–42_ was measured by enzyme-linked immunosorbent assay (ELISA; Thermo Fisher Scientific, Inc., Waltham, MA, USA). To assess the capacity for Aβ_1–42_ degradation, BV2 microglia were incubated with 5 μM Aβ_1–42_ for 6 h first (t = 6 h), washed with fresh medium, and maintained for an additional 6 h in serum- and Aβ_1–42_-free conditions (t = 12 h). Afterward, the cells were trypsinized and lysed for ELISA. The Aβ_1–42_ degradation index was expressed as the total amount of internalized Aβ_1–42_ (t = 6 h)/remaining Aβ_1–42_ (t = 12 h) ratio. A higher degradation index suggests a better capacity for degradation of Aβ_1–42_.

### 2.4. Western Blot Analysis

Western blot analysis was carried out as described previously [20]. Total protein was extracted from BV2 microglia. Equal amounts of protein were separated on sodium dodecyl sulfate polyacrylamide gels, transferred to polyvinylidene fluoride membranes, and blocked with 5% bull serum albumin for 1 h at 25 °C. Membranes were incubated overnight with an antibody against iNOS (1:800, Abcam, Inc., Boston, MA, USA), an antibody against ARG1 (1:800, Abcam, Inc., Boston, MA, USA), an antibody against CD206 (1:1200, Abcam, Inc., Boston, MA, USA), or an antibody against β-actin (1:1200, Cell Signaling Technology, Inc., Danvers, MA, USA), then washed and incubated with horseradish peroxidase-coupled secondary antibodies for 2 h at 25 °C. The protein bands were detected by chemiluminescence (BioRad, Inc., Hercules, CA, USA), and their optical density was measured using Quantity One software (BioRad, Inc., Hercules, CA, USA). Relative protein levels were normalized to β-actin.

### 2.5. ELISA

ELISA was carried out as described previously [20]. The culture medium of BV2 microglia was collected and centrifuged at 1000 × *g* for 15 min to remove cellular debris. The levels of the inflammatory cytokines, including interleukin (IL)-1β (Abcam, Inc., Boston, MA, USA), IL-6 (Abcam, Inc., Boston, MA, USA), and tumor necrosis factor (TNF)-α (Abcam, Inc., Boston, MA, USA), were detected by specific detection kits according to the manufacturer’s protocols.

### 2.6. Statistical Analysis

The statistical analysis was carried out using GraphPad Prism software (GraphPad Software, Inc., San Diego, CA, USA), as described previously [20]. One-way ANOVA followed by Tukey’s post hoc test was used to analyze differences among three groups. The Student’s *t*-test was employed for comparisons between two groups. Data are expressed as the means ± SDs. *p* < 0.05 was considered statistically significant.

## 3. Result

### 3.1. The Trem2 H157Y Variant Inhibits Microglial Phagocytosis of Aβ

**Figure 1:**
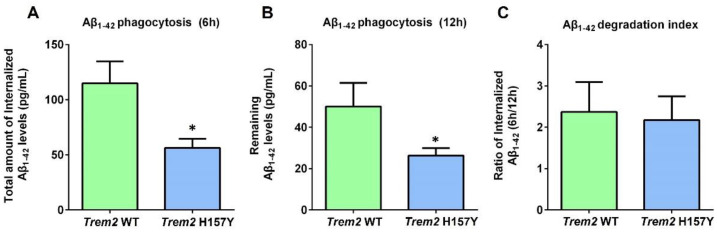
The *Trem2* H157Y variant inhibits microglial phagocytosis of Aβ. An in vitro assay was employed to evaluate microglial Aβ_1–42_ phagocytosis and degradation. (**A**) WT *Trem2* BV2 microglia or BV2 microglia with the *Trem2* H157Y variant were incubated with 5 μM Aβ_1–42_ for 6 h. The amount of internalized Aβ_1–42_ was measured by ELISA (t = 6 h). (**B**) Later, the Aβ_1–42_ left in the medium was washed out, and WT *Trem2* BV2 microglia or BV2 microglia with the *Trem2* H157Y variant were maintained for an additional 6 h to allow for Aβ_1–42_ degradation. The intracellular Aβ_1–42_ levels were assessed by ELISA again (t = 12 h). (**C**) The index of Aβ_1–42_ degradation was expressed as the ratio of the total amount of internalized Aβ_1–42_ (t = 6 h) to remaining Aβ_1–42_ (t = 12 h). A higher degradation index indicates a better capacity for degradation of Aβ_1–42_. Data were analyzed by one-way ANOVA followed by Tukey’s post hoc test. Columns represent means ± SDs (n = 3, performed in triplicates), * *p* < 0.05 versus WT *Trem2* BV2 microglia.

Given that microglial phagocytosis and degradation of Aβ plays a crucial role in AD progression [5], we employed an in vitro assay to investigate the influence of the *Trem2* H157Y variant on microglial Aβ phagocytosis and degradation. As revealed in Figure 1A, BV2 microglia with the *Trem2* H157Y variant showed a 51.1% decrease in internalized Aβ_1–42_ levels when compared with WT *Trem2* BV2 microglia. It is worth noting that no significant difference was observed in Aβ_1–42_ degradation indexes between BV2 microglia with the *Trem2* H157Y variant and WT *Trem2* BV2 microglia (Figure 1C).

### 3.2. The Trem2 H157Y Variant Promotes M1-Type Polarization of Microglia

**Figure 2:**
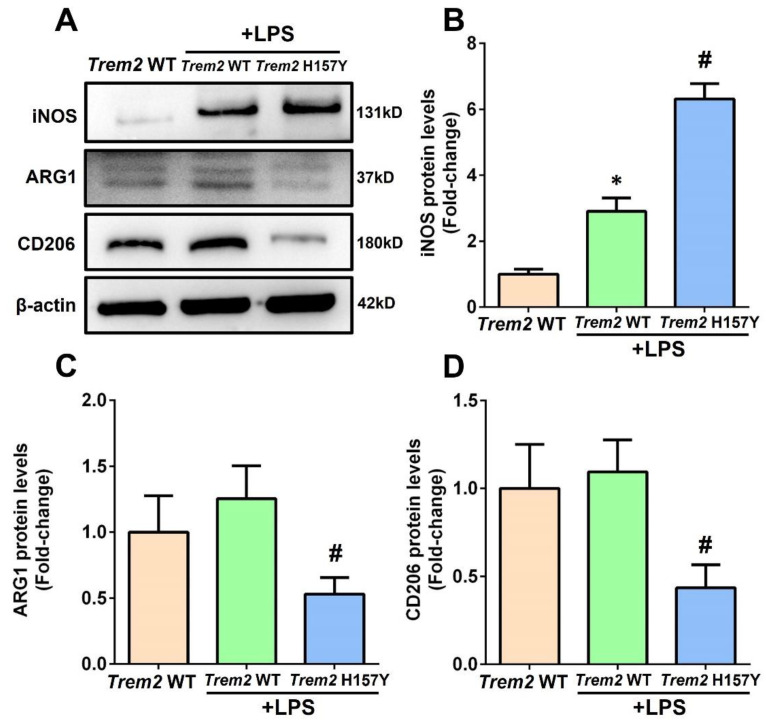
The *Trem2* H157Y variant promotes M1-type polarization of microglia. (**A**) Levels of iNOS, ARG1, and CD206 in WT *Trem2* BV2 microglia and BV2 microglia with the *Trem2* H157Y variant after LPS stimulation were detected by Western blot analysis. (**B**–**D**) Quantitative analysis of iNOS, ARG1, and CD206 protein levels. Data were normalized to β-actin. Data were analyzed by one-way ANOVA followed by Tukey’s post hoc test. Columns represent means ± SDs (n = 3, performed in triplicates). * *p* < 0.05 versus WT *Trem2* BV2 microglia without LPS stimulation; # *p* < 0.05 versus LPS-stimulated WT *Trem2* BV2 microglia.

After stimulation, microglia showed either pro-inflammatory status (M1-type polarization) or anti-inflammatory status (M2-type polarization) [23]. To explore the influence of the *Trem2* H157Y variant on microglial polarization, BV2 microglia were stimulated with 100 ng/mL LPS. As revealed in Figure 2A, B, LPS stimulation increased the levels of iNOS, a marker of M1-type polarization (*p* < 0.05) [24], in WT *Trem2* BV2 microglia. Contrastingly, BV2 microglia with the *Trem2* H157Y variant exhibited significantly higher levels of iNOS after LPS stimulation. As indicated in Figure 2A, C, D, LPS stimulation increased the protein levels of ARG1 and CD206, two markers of M2-type polarization [25], in WT *Trem2* BV2 microglia, but these increments did not reach statistical significance (*p* > 0.05). In contrast, BV2 microglia with the *Trem2* H157Y variant showed reduced protein levels of ARG1 and CD206 following LPS stimulation.

### 3.3. The Trem2 H157Y Variant Facilitates Microglial Release of Inflammatory Cytokines

**Figure 3:**
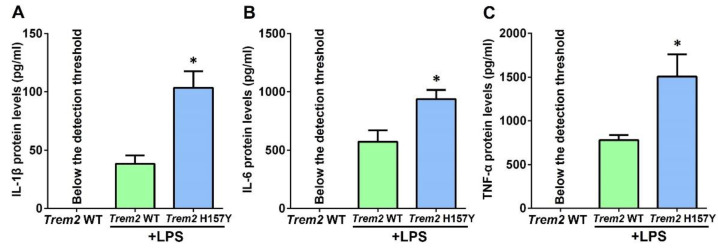
The *Trem2* H157Y variant facilitates microglial release of inflammatory cytokines. (**A**–**C**) Levels of the inflammatory cytokines IL-1β, IL-6, and TNF-α in WT *Trem2* BV2 microglia and BV2 microglia with the *Trem2* H157Y variant were detected by ELISA. Data were analyzed using the Student’s *t*-test. Columns represent means ± SDs (n = 3, performed in triplicates). * *p* < 0.05 versus LPS-stimulated WT *Trem2* BV2 microglia.

Afterward, we investigated the influence of the *Trem2* H157Y variant on microglial inflammatory cytokine release. Stimulating WT *Trem2* BV2 microglia with LPS led to release of IL-1β, IL-6, and TNF-α into the culture medium. As revealed in Figure 3A–C, BV2 microglia with the *Trem2* H157Y variant released more inflammatory cytokines, including IL-1β, IL-6, and TNF-α, than WT *Trem2* BV2 microglia after LPS stimulation.

## 4. Discussion

*TREM2* is a newly identified susceptibility gene for AD [26]. In Caucasians, a rare coding variant, R47H, was reported to increase the risk of AD with an odds ratio of 4.7 [27,28]. Meanwhile, we identified that another rare coding variant H157Y substantially elevated AD risk in a Han Chinese population [14]. To date, how the *TREM2* H157Y variant increases AD risk has not been fully understood. Human *TREM2* encodes a 230-amino acid type I transmembrane receptor, which is exclusively expressed in microglia in the brain [29]. It contains an Ig-like V-type domain, a stalk region, a transmembrane domain, and a short cytoplasmic tail [30]. An increasing amount of evidence suggested that full-length TREM2 participated in AD progression via facilitated microglial phagocytosis of Aβ and suppressed M1-type polarization of microglia [22,31]. In this study, we have provided the first evidence that the *Trem2* H157Y variant inhibits microglial Aβ phagocytosis. This finding was supported by a recent study from Schlepckow and colleagues showing that the *TREM2* H157Y variant impaired phagocytosis of *E.coli* pHrodo particles in HEK293 cells [32]. Moreover, in the current study, we observed that the M1-type polarization marker iNOS was increased while the M2-type polarization markers ARG1 and CD206 were reduced in BV2 microglia with the *Trem2* H157Y variant. To our knowledge, this is the first study reporting that the *Trem2* H157Y variant induced microglial M1-type polarization. Under physiological conditions, a disintegrin and metalloproteinase (ADAM) 10/17 cleaves the full-length TREM2 protein at the H157-S158 site [33]. In HEK293 cells, the *TREM2* H157Y variant was revealed to facilitate ADAM10/17 cleavage and thus reduce membrane-associated full-length TREM2 protein levels [32]. In view of the above evidence, the M1-type microglial polarization and impaired microglial Aβ phagocytosis caused by the H157Y variant might be ascribed to the reduction in full-length TREM2 protein levels on the surface of microglia.

Another interesting finding of this study was that the *Trem2* H157Y variant promoted microglial release of inflammatory cytokines. The *TREM2* H157Y variant was reported to enhance sTREM2 production via enhanced ADAM10/17 cleavage [33]. As a bioactive fragment of full-length TREM2 protein, sTREM2 was reported to promote microglial inflammatory cytokine release and subsequently induce neuroinflammation [34]. Therefore, the elevated inflammatory cytokine release caused by the *Trem2* H157Y variant might be attributed to the enhanced sTREM2 production. However, our findings appeared to contradict those of a recent study by Qiao and colleagues [35], as they found that the *Trem2* H157Y variant downregulated neuroinflammation-related genes in *Trem2* H157Y knock-in 5xFAD transgenic mice. It should be noted that our experiments were conducted in a cellular inflammation model induced by LPS, whilst Qiao et al. employed an animal model of amyloid pathology to validate the function of the *Trem2* H157Y variant [35]. The pathophysiological bases underlying these two models are quite different, which may account for these opposite observations.

This study also had some limitations. First, the impacts of the H157Y variant on the TREM2 downstream signaling pathway were not investigated in this study. In the future, the main components of the TREM2 downstream signaling pathway, such as TYROBP and β-catenin in microglia with the *Trem2* H157Y variant, should be assessed [36,37,38]. Second, in this study, the functions of the H157Y variant were investigated using mouse BV2 microglial cells. Although TREM2 protein seemed to be conserved across mammals, our findings should be further confirmed using human microglia.

## 5. Conclusions

Summarily, in the current study, using CRISPR-Cas9-engineered BV2 microglia, we provide the first evidence that the *Trem2* H157Y variant inhibits microglial phagocytosis of Aβ, promotes M1-type polarization of microglia, and facilitates microglial release of inflammatory cytokines. These findings provide new insights into the cellular mechanisms by which the *TREM2* H157Y variant elevates the risk of AD.

## Data Availability

The data that support the findings of this study are available from the corresponding author upon reasonable request.
